# Effects of Bioactive Microalgal Pigment Intake on Metabolic Parameters, Cecal Distribution and Short-Chain Fatty Acids in a Diabetic Rat Model

**DOI:** 10.3390/life16091540

**Published:** 2026-09-15

**Authors:** Patrícia A. Caetano, Tatiele C. do Nascimento, Marcylene V. da Silveira, Duane S. Vieira, Luiz Eduardo Lobo, Maria R. C. Schetinger, Cristiano R. de Menezes, Roger Wagner, María Roca, Eduardo Jacob-Lopes, Leila Q. Zepka

**Affiliations:** 1 Food Science and Technology Department, Federal University of Santa Maria, Santa Maria 97105-900, RS, Brazil; 2 Department of Biochemistry and Molecular Biology, Federal University of Santa Maria, Santa Maria 97105-900, RS, Brazil; 3 Food Phytochemistry Department, Instituto de la Grasa (CSIC), University Campus, Building 46, 41013 Sevilla, Spain; roca@ig.csic.es

**Keywords:** carotenoids, chlorophylls, microalgae, short-chain FATTY acids, glycemia, T2DM

## Abstract

Type 2 diabetes mellitus (T2DM) represents a major global public health challenge, and bioactive compounds have been investigated as complementary nutritional strategies for disease management. This study evaluated, for the first time, the effects of carotenoids and chlorophylls (lipophilic extract) of *Scenedesmus obliquus* on the cecal pigment profile, short-chain fatty acids (SCFAs), metabolic parameters, and intestinal fate in an experimental model of T2DM. Healthy and diabetic Wistar rats were supplemented for 28 days with the lipophilic extract (300 mg/kg body weight), alone or in combination with metformin (150 mg/kg bw). Chlorophyll *a* and all-*trans*-lutein were the predominant in the extract, whereas all-*trans*-lutein and chlorophyll derivatives predominated in the cecal contents. β-Carotene-5,6,5′,8′-diepoxide and porphyrins were detected exclusively in the cecum, indicating extensive metabolic transformations during gastrointestinal digestion. Supplementation significantly reduced blood glucose levels and favorably modulated the SCFA profile, restoring the acetate-to-propionate ratio to values comparable to those of healthy animals. Although total cecal pigment content did not differ among groups, carotenoids and chlorophyll derivatives exhibited distinct accumulation patterns, suggesting differential intestinal metabolism. Overall, the findings indicate that the host metabolic status modulates the intestinal fate of microalgal pigments, supporting their potential contribution to glycemic regulation and intestinal fermentation in T2DM.

## 1. Introduction

Over the past decades, type 2 diabetes mellitus (T2DM) has emerged as a major global public health challenge. According to estimates from the World Health Organization (WHO), the disease affects approximately 176 million people worldwide, and one diabetes-related death occurs every 10 s [1]. Although treatment is predominantly based on pharmacological interventions combined with the adoption of a healthy lifestyle [2], nutrition has emerged as an important complementary non-pharmacological strategy for the management of metabolic disorders [3]. In parallel, approximately 70% of the global population relies on natural therapies, including herbal infusions and plant extracts, to treat or prevent diseases [1]. In this context, microalgae have emerged as a sustainable source of bioactive compounds, providing extracts rich in carotenoids and chlorophylls that have increasingly been incorporated into functional foods and dietary supplements because of their potential health-promoting properties [4].

Since these pigments cannot be synthesized by the human body, their availability depends entirely on dietary intake. Several studies have demonstrated promising results regarding the bioaccessibility and bioavailability of carotenoids and chlorophylls derived from microalgae, particularly when administered as isolated extracts [5,6,7,8,9,10]. Consistent evidence indicates that these compounds exhibit strong antioxidant capacity and possess antimicrobial, antimutagenic, and antigenotoxic activities, while also contributing to cancer risk reduction, prevention of lipid oxidation, and regulation of body weight gain [11,12,13].

However, a recurring finding in these bioavailability studies deserves attention: a substantial proportion of the bioaccessible pigments generated during digestion is not absorbed by intestinal cells. This observation raises important questions regarding their metabolic fate, potential transformation pathways, and biological effects beyond systemic circulation. Recently, Nascimento et al. [14] demonstrated that carotenoids and chlorophylls derived from *Scenedesmus obliquus* are extensively distributed throughout the cecum, a region of the large intestine characterized by intense fermentative activity. Furthermore, supplementation with different doses of this extract was associated with increased production of short-chain fatty acids (SCFAs) in healthy rats.

Under pathological conditions, recent evidence indicates that T2DM is closely associated with dysregulated SCFA production. These metabolites play a pivotal role in maintaining host homeostasis by regulating intestinal barrier integrity, inflammatory responses, and glucose metabolism [15]. Consequently, the gut has emerged as an important therapeutic target for nutritional and pharmacobiotic interventions [16], stimulating the search for strategies capable of restoring or preserving host homeostasis through modulation of the intestinal microbiome [15,17,18,19,20].

Supporting this concept, Paul et al. [21] demonstrated an association between a microalgae-derived chlorophyll metabolite and class I glucose transporters, particularly GLUT1 and GLUT4, in pancreatic β-cells, suggesting enhanced glucose-stimulated insulin secretion and improved glycemic homeostasis. In addition, different microalgal extracts evaluated in vitro inhibit digestive enzymes that catalyze carbohydrate hydrolysis, such as α-amylase, thereby delaying carbohydrate digestion and glucose absorption and consequently reducing postprandial hyperglycemia [22,23,24]. These effects have been attributed, at least in part, to the presence of bioactive compounds, particularly carotenoids, whose antioxidant, anti-inflammatory, and anti-apoptotic properties may protect against metabolic alterations associated with T2DM [22,25]. Thus, these findings suggest that microalgae-derived compounds may contribute to T2DM management through multiple mechanisms, such as maintaining redox balance and modulating the inflammatory response, improving insulin sensitivity, and regulating carbohydrate digestion and absorption.

Collectively, these findings indicate that microalgae-derived compounds represent a promising complementary nutritional strategy for T2DM management, not only by modulating metabolic alterations but also through their potential action along the gut–metabolism axis [21,26]. Nevertheless, it remains unclear how carotenoid- and chlorophyll-rich extracts simultaneously influence the intestinal distribution of these pigments, the fermentative activity of the gut microbiota, and their relationship with metabolic parameters in experimental models of T2DM.

Therefore, this study investigated, for the first time, the effects of a lipophilic pigment extract from *S. obliquus* on the cecal distribution, the profile of SCFAs, and metabolic parameters in rats with T2DM. Unlike conventional approaches that focus exclusively on systemic glycemic markers, this study integrates carotenoid and chlorophyll characterization with intestinal metabolism, providing new insights into the potential interactions among microalgal pigments, cecal fermentation, and glycemic control.

## 2. Materials and Methods

### 2.1. Chemicals and Reagents

HPLC-grade solvents were sourced from VWR BDH Chemicals (Radnor, PA, USA). Pigment standards and additional analytical-grade reagents were purchased from Sigma-Aldrich Chemical Co. (Madrid, Spain). All reagents and solvents used in the experimental part were of analytical purity.

### 2.2. Microalgal Culture and Biomass Production

Monocultures of *S. obliquus* (CPCC05) were obtained from the Canadian Phycological Culture Centre (CPCC), University of Toronto, Toronto, Canada. Culture stocks were propagated on agar-solidified (20 g/L) BG-11 [27]. Biomass production was carried out under phototrophic conditions in a bubble column photobioreactor (Tecnal, Piracicaba, SP, Brazil) supplied with 2.0 L of BG-11 medium and operated in batch mode [28]. The cultivation was carried out until the stationary phase, when biomass density reached its maximum. Following cultivation, the biomass was recovered from the culture medium by centrifugation (1500× *g*, 10 min, 10 °C) (model D-37520, Thermo, Langenselbold, Germany). The biomass paste was first maintained at −18 °C for 24 h and subsequently freeze-dried at −50 °C for 24 h under reduced pressure (<175 μm Hg).

### 2.3. Microalgal Pigment Extraction

Pigments were exhaustively extracted from 10 mg of *S. obliquus* biomass. Initially, 50 mL of dimethylformamide (DMF) saturated with magnesium carbonate was added to the biomass. The suspension was subjected to ultrasonication, vortex mixing, and centrifugation, after which the supernatant was collected. The remaining pellet was subsequently extracted with 50 mL of methanol using the same sequence of ultrasonication, vortex mixing, centrifugation, and supernatant collection. The extraction procedure was repeated until no additional pigments were recovered. The combined extracts were transferred to a separatory funnel containing 150 mL of diethyl ether and 200 mL of 10% (*w*/*v*) sodium chloride (NaCl) solution. After vigorous mixing and complete phase separation, the organic phase was collected and washed three consecutive times with 200 mL of the NaCl solution. The solvent was then removed under reduced pressure using a rotary evaporator at 30 °C until complete solvent evaporation was achieved. Finally, the resulting residue was reconstituted in 1 mL of acetone, and passed through a 0.22 μm nylon membrane filter for chromatographic analysis.

### 2.4. Pigments Identification by HPLC-ESI/APCI-HRQqTOF-MS2

Chromatographic analyses were carried out on a Dionex Ultimate 3000 RS UHPLC system (Thermo Fisher Scientific, Waltham, MA, USA). Separations were achieved using a Mediterranea Sea18 analytical column (200 × 4.6 mm, 3 μm; Teknokroma, Barcelona, Spain) coupled to a guard column (10 × 4.6 mm) packed with the same stationary phase. Pigments were eluted according to the gradient previously described [29], employing two mobile phases: phase A consisting of water/1 M ammonium acetate in water/methanol (1:1:8, *v*/*v*/*v*) and phase B composed of methanol/acetone (1:1, *v*/*v*). UV–visible absorption spectra were continuously acquired between 350 and 800 nm using a photodiode array detector. Mass spectrometric characterization was performed with a micrOTOF-QII high-resolution quadrupole time-of-flight mass spectrometer (Bruker Daltonics, Bremen, Germany) hyphenated to the UHPLC system. Electrospray ionization (ESI) was applied for the detection of polar pigments, whereas atmospheric pressure chemical ionization (APCI) was used for non-polar compounds.

### 2.5. Pigment Quantification by HPLC-PDA-UV-Visible

Pigment quantification was carried out by reverse-phase HPLC using an Agilent 1260 liquid chromatography system following the chromatographic conditions described above. Chromatographic data were acquired and processed with OpenLab CDS software (version C.01.07; Agilent Technologies, Santa Clara, CA, USA). Detection was performed sequentially at wavelengths of 410, 430, 450, 650, and 666 nm. Quantitative determination of each compound was based on external calibration curves generated by plotting peak area against analyte concentration. Calibration equations were established by least-squares linear regression using concentration ranges representative of the pigment levels detected in the samples. Five injection volumes of each standard solution were analyzed in triplicate to construct the calibration curves.

### 2.6. In Vivo Experimental Design

Sixty male Wistar rats (200–250 g; ~45 days) were obtained from the Animal Reproduction Center of the Federal University of Santa Maria and randomly distributed into six groups (*n* = 10): three healthy—SSH (saline solution–Healthy); LEH (lipophilic extract–Healthy); MLEH (metformin [150 mg/g] + lipophilic extract–Healthy); and three diabetic—SSD (saline solution–Diabetic); LED (lipophilic extract–Diabetic); MLED (metformin [150 mg/g + lipophilic extract–Diabetic). The animals were kept at 23 ± 1 °C, in a 12 h light/dark cycle, with commercial diet (CD) (Puro Lab 22 PB^®^, Puro Trato, Brazil) and a 15-day adaptation period. Baseline blood glucose was measured in the caudal vein (Accu-Chek^®^, Mannheim, Germany). All procedures followed ethical guidelines (protocol no. 2662070222).

T2DM was induced by the combined administration of a high-lipid-density diet (HLDD) and a low dose of streptozotocin (STZ), following the protocol described by Srinivasan et al. [30]. Animals assigned to the T2DM groups received an HLDD containing 15% sucrose, 10% lard, and 5% egg for a total of 10 weeks. After 5 weeks of HLDD feeding, the animals were fasted for 6 h, blood glucose levels were measured, and a single dose of STZ (35 mg/kg) was administered intraperitoneally in 0.1 M sodium citrate buffer (pH 4.5). Three days after STZ administration, blood glucose levels were measured to confirm diabetes induction, and animals with blood glucose > 250 mg/dL were considered diabetic and included in the study. Following diabetes confirmation, the animals continued to receive the HLDD until completion of the 10-week dietary period. Thus, diabetes induction resulted from the combined HLDD–STZ protocol rather than from the HLDD alone. The control group received a standard diet and citrate buffer without STZ. Animals that did not meet the predefined criteria for diabetes induction were excluded before the intervention in accordance with the established ethical criteria.

The dosage of the lipophilic extract (300 µg/kg.bw), defined based on Nascimento et al. [14], was administered for 28 days, starting 10 weeks after the induction of diabetes (HLDD + STZ). The lipophilic pigment extract was suspended in 300 µL of sunflower oil and administered via oral gavage. The other groups underwent the same procedure but without the pigments. At the end of the experiment, after a period of 6 h, the animals were anesthetized (isoflurane) and subjected to euthanasia for blood collection (for determination of biochemical parameters) and cecum (for determination of pigments and SCFAs).

#### 2.6.1. Determination of Body Weight and Blood Glucose Levels

Throughout the experimental period, body weight and blood glucose concentrations were recorded at four scheduled time points: (I) after adaptation; (II) after 5 weeks of HLDD; (III) after T2DM induction with STZ; and (IV) after treatment. During the 28-day experimental period, body weight was measured at approximately 3-day intervals.

#### 2.6.2. Determination of Pigments in Diets and Cecal Samples

To minimize pigment photo-oxidation, all extraction procedures were conducted under dim green light. Commercial diet (100 mg), HLDD (100 mg), and cecal content (50 mg) samples were homogenized in 50 mL of DMF saturated with Mg_2_CO_3_ and subsequently vacuum-filtered. The retained solid material was subjected to two additional extraction cycles under identical conditions. Extracts obtained from the initial and subsequent extraction steps were pooled in a separatory funnel containing 150 mL of diethyl ether and 200 mL of 10% (*w*/*v*) NaCl solution. After thorough mixing, the system was left undisturbed until complete separation of the organic and aqueous phases was achieved. The organic phase was recovered and evaporated to dryness under reduced pressure at 30 °C using a rotary evaporator. Pigment reconstitution, identification and quantification were carried out as described in Section 2.4 and Section 2.5.

#### 2.6.3. Determination of SCFA

For short-chain fatty acid (SCFA) determination, cecal samples were thawed at 5 °C and manually homogenized before analysis. Aliquots (1 g) were transferred to 2 mL polypropylene microcentrifuge tubes and centrifuged at 12,300× *g* for 5 min. Subsequently, 250 μL of the clarified supernatant was mixed with an equal volume of formic acid in a clean microtube. After manual homogenization, the mixture was centrifuged again for 3 min. An aliquot (250 μL) of the resulting supernatant was transferred to a chromatographic vial, followed by the addition of 500 μL of a 3-octanol solution (665 μg/mL in methanol), which served as the internal standard. The final mixture was homogenized prior to injection.

SCFAs were analyzed using a gas chromatograph equipped with a flame ionization detector (GC-FID; Varian Star 3400, Palo Alto, CA, USA) and an autosampler (Varian 8200CX, Palo Alto, CA, USA). A 1 μL aliquot of each sample was injected in split mode (1:10). Hydrogen was used as the carrier gas at a constant pressure of 20 psi. Separation of acetic, propionic, and butyric acids was achieved on a CP-WAX 52CB capillary column (60 m × 0.25 mm, 0.25 μm film thickness). The oven temperature was initially maintained at 80 °C for 1 min, increased to 120 °C at 8 °C/min, and then raised to 230 °C at 20 °C/min, where it was held for an additional minute. Both injector and detector temperatures were maintained at 250 °C. SCFA concentrations were expressed as mol per 100 mL (mol/100 mL).

### 2.7. Statistical Analysis

All statistical procedures were performed in Statistica 10.0 (StatSoft Inc., Tulsa, OK, USA). Comparisons among groups were carried out using one-way ANOVA, and significant differences were identified by Tukey’s post hoc test (*p* < 0.05). Multivariate analysis was further explored by principal component analysis (PCA) using the same statistical software. Results are expressed as mean values ± standard deviation (SD).

## 3. Results and Discussion

### 3.1. Body Weight and Blood Glucose Levels

**Figure 1:**
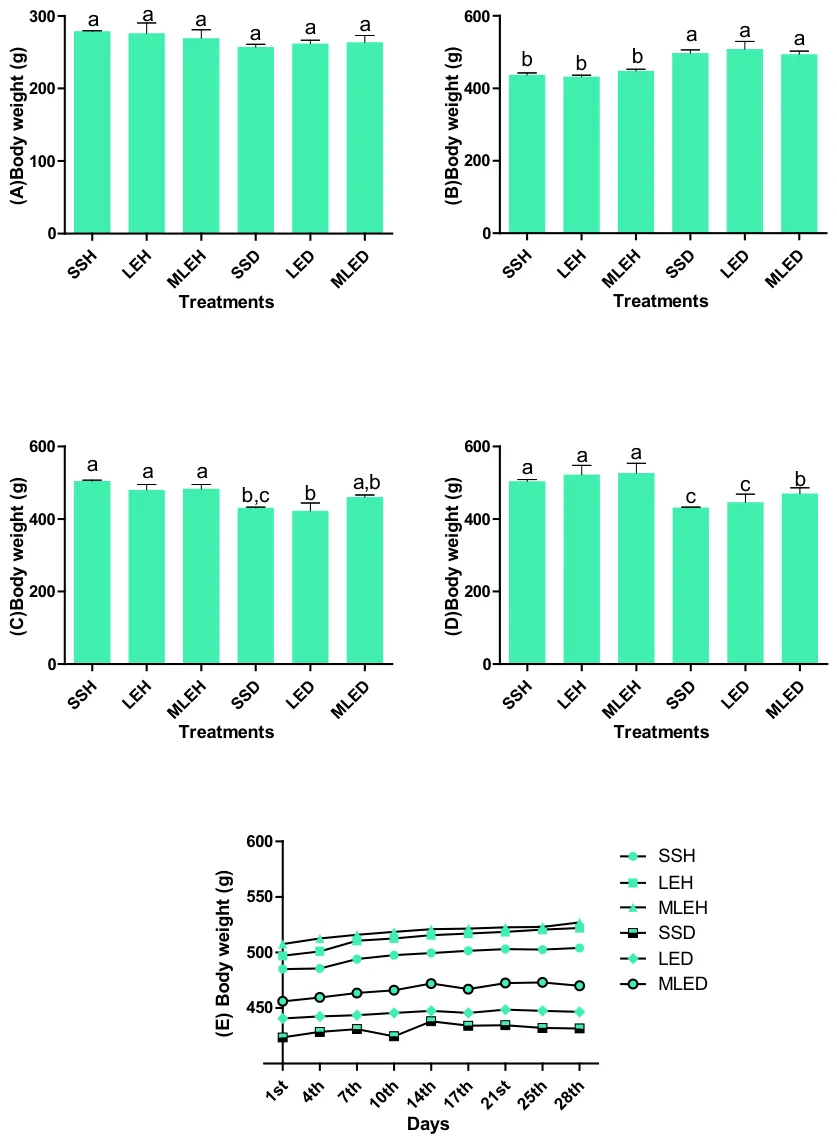
Body weight of the experimental groups at different stages: after adaptation (**A**); 5 weeks after HLDD administration (**B**); induction of diabetes (**C**); after treatment (**D**); changes in body weight over the 28-day experiment (**E**). SSH: saline solution–Healthy; LEH: lipophilic extract–Healthy; MLEH: metformin + lipophilic extract–Healthy; SSD: saline solution–Diabetic; LED: lipophilic extract–Diabetic; MLED: metformin + lipophilic extract–Diabetic. Different letters between columns indicate statistical differences (*p* < 0.05).

**Figure 2:**
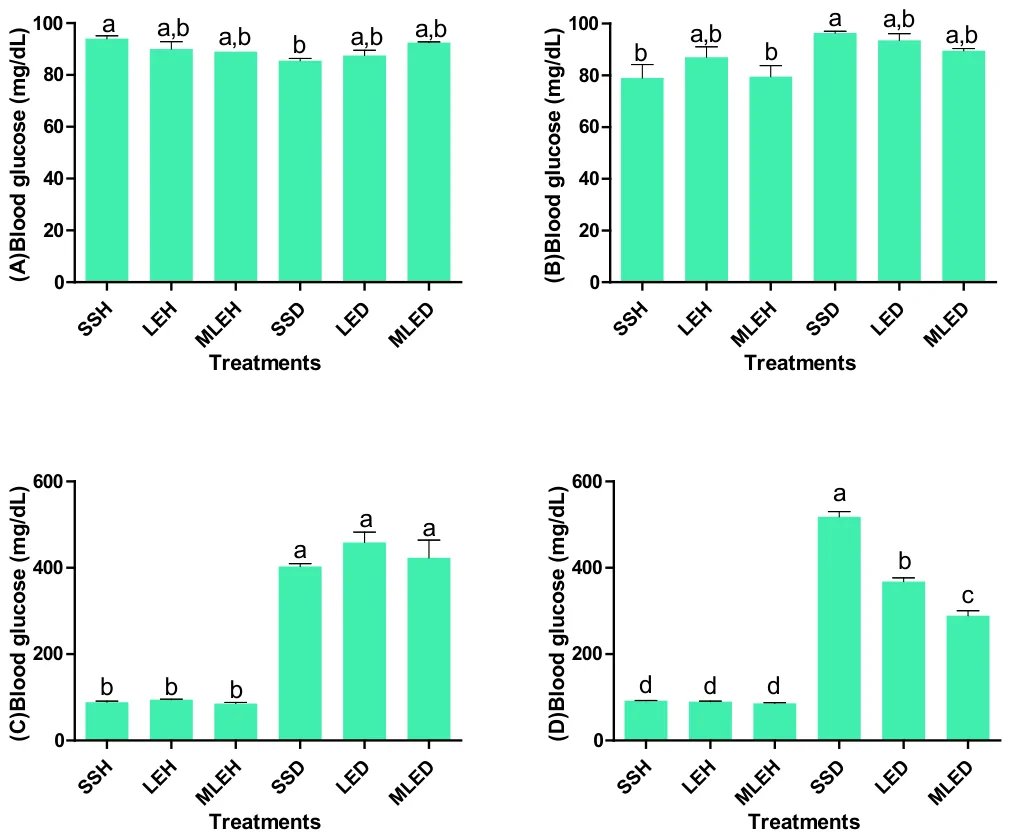
Glucose levels of the experimental groups after adaptation—before HLDD (**A**), 5 weeks of HLDD administration (**B**), induction of diabetes (**C**), and end of the experiment—after treatment (**D**). SSH: saline solution–Healthy; LEH: lipophilic extract–Healthy; MLEH: metformin + lipophilic extract–Healthy; SSD: saline solution–Diabetic; LED: lipophilic extract–Diabetic; MLED: metformin + lipophilic extract–Diabetic. Different letters between columns indicate statistical differences (*p* < 0.05).

Figure 1 and Figure 2 present the body weight and blood glucose levels of the experimental groups, respectively. As shown in Figure 1A, no statistically significant differences in body weight were observed among the groups after the acclimatization period, indicating comparable baseline characteristics across all animals. After five weeks of the HLDD, SSD, LED, and MLED groups exhibited significantly greater body weight than the SSH, LEH, and MLEH groups (Figure 1B). This outcome was expected, as the former groups were fed the hypercaloric diet, whereas the latter received the standard commercial diet.

Considering the effect of diabetes induction on body weight (Figure 1C), the healthy groups (SSH, LEH, and MLEH) exhibited the highest body weights. Conversely, the diabetic groups SSD and LED showed a significant reduction in body weight, while the MLED group presented intermediate values (*p* < 0.05). This trend persisted until the end of the experiment; however, the combined treatment with the lipophilic extract and standard medication (MLED) (Figure 1D) significantly increased body weight compared with the untreated diabetic group (SSD). Overall, this profile was also observed throughout the 28-day experimental period (Figure 1E).

The reduction in body weight observed in diabetic animals may be associated with a compensatory mechanism for energy supply in type 2 diabetes mellitus, in which the organism mobilizes fat reserves and skeletal muscle protein through increased lipolysis and gluconeogenesis, respectively, resulting in loss of body mass [31,32].

Regarding glucose levels, at baseline (Figure 2A), the groups showed similar blood glucose values, with no statistically significant differences among SSH, LEH, MLEH, LED, and MLED (*p* > 0.05). The SSD group presented slightly lower values, although still within the normoglycemic range [33].

The groups fed a hypercaloric diet (SSD, LED, and MLED) showed slightly higher blood glucose levels than those fed a standard diet (SSH, LEH, and MLEH) (Figure 2B). Additionally, the SSD group presented significantly higher values compared with the SSH and MLEH groups (*p* < 0.05). However, blood glucose levels did not exceed 100 mg/dL.

After diabetes induction (Figure 2C), a marked difference between healthy and diabetic animals was observed. The healthy groups (SSH, LEH, and MLEH) exhibited significantly lower blood glucose levels, whereas the diabetic groups (SSD, LED, and MLED) showed pronounced hyperglycemia (*p* < 0.05). Considering the coexistence of weight loss (Figure 2C) and hyperglycemia, this condition is consistent with a catabolic state associated with impaired insulin action, characterized by the mobilization of energy reserves, along with glycosuria and increased gluconeogenesis [34]. However, the lack of serum insulin, glucose intolerance test (GTT), insulin tolerance test (ITT), and glycated hemoglobin assessments limits a more comprehensive characterization of glucose homeostasis and precludes direct conclusions regarding insulin resistance or sensitivity.

At the end of the experiment, following the administration of treatments (Figure 2D), the untreated diabetic group (SSD), as well as the groups treated with the lipophilic extract (LED) or with metformin combined with the extract (MLED), maintained elevated blood glucose levels compared to the healthy control groups (*p* < 0.05). However, the LED- and MLED-treated diabetic groups showed significant reductions of 29% and 44%, respectively, compared to the SSD group (*p* < 0.05). Consistent with this, the SSD group exhibited the highest serum levels of aspartate aminotransferase (AST) (177.80 U/L) and alanine aminotransferase (ALT) (90.10 U/L) (see Table S1). Lower values of AST (145.60 and 138.90 U/L, respectively) and ALT (70.7 and 45.55 U/L, respectively) were observed in the LED and MLED groups. Notably, in the MLED group, ALT levels did not differ significantly from those observed in the healthy groups. These findings indicate a concomitant improvement in the biochemical parameters evaluated in the treated animals, although AST and ALT levels alone are insufficient to confirm hepatic injury or protection.

Taken together, the body weight and glucose results demonstrate that a hypercaloric diet combined with chemical diabetes induction promoted consistent metabolic alterations, characterized by initial weight gain followed by weight loss and marked hyperglycemia in diabetic groups. Administration of the microalgal lipophilic extract, particularly when combined with metformin, partially attenuated these alterations, promoting recovery of body weight and a significant reduction in glycemia compared with the untreated diabetic group. In agreement with previous studies [21,25,35,36], these results indicate that the administration of carotenoids and chlorophyll, either alone or in combination with metformin, reinforces their potential as a complementary strategy to conventional therapy.

### 3.2. Carotenoid and Chlorophyll Identification by HPLC-PDA-MS/MS

Table 1 compiles the chromatographic and mass spectrometric characteristics of the carotenoids and chlorophylls identified throughout the present study. Pigment identification was based on chromatographic elution order, UV–visible absorption spectra, and high-resolution mass spectrometry data, including mass error, isotopic pattern, and characteristic product ions. These data were compared with authentic standards or compounds previously isolated from natural sources and further corroborated by descriptions available in the literature [9,29,37].

A total of twenty-eight compounds were separated, of which sixteen were identified in the *S. obliquus* extract. These comprised seven carotenoids—all-*trans*-neoxanthin (peak 7), *cis*-lutein (peak 8), all-*trans*-violaxanthin (peak 9), all-*trans*-lutein (peak 12), 9-*cis*-lutein (peak 13), 9-*cis*-zeaxanthin (peak 17), and all-*trans*-β-carotene (peak 27)—and nine chlorophyll derivatives: chlorophyllide *a* (peak 1), pheophorbide *a* (peak 6), chlorophyll *b* (peak 14), chlorophyll *b′* (peak 15), 13^2^-OH-chlorophyll *a* (peak 16), chlorophyll *a* (peak 18), chlorophyll *a′* (peak 19), pheophytin *a* (peak 25), and pheophytin *a′* (peak 26). This pigment composition is characteristic of green microalgae and is consistent with previous reports for *Chlorophyceae* [9,37].

Fifteen compounds were detected in the commercial diet (CD), including six carotenoids (*cis*-lutein, 13- or 15-*cis*-zeaxanthin, all-*trans*-lutein, 9-*cis*-lutein, 9-*cis*-zeaxanthin, and all-*trans*-β-carotene) and ten chlorophyll derivatives. The hypercaloric diet (HLDD) exhibited a similar pigment profile, differing only by the absence of 13^2^-OH-pheophorbide *a*. The occurrence of pheophorbides (peaks 4 and 6), hydroxypheophytins (peaks 20–22 and 24), pheophytins (peaks 23, 25, and 26), and pyropheophytin (peak 28) is consistent with degradation processes resulting from pheophytinization, hydroxylation, and pyrolysis during feed processing [38,39].

Chromatographic analysis of the cecal samples revealed the presence of eighteen distinct compounds across the experimental groups. Among the carotenoids, β-carotene-5,6,5′,8′-diepoxide (peak 11), all-*trans*-lutein (peak 12), 9-*cis*-lutein (peak 13), 9-*cis*-zeaxanthin (peak 17), and all-*trans*-β-carotene (peak 27) were detected in all groups, except for β-carotene-5,6,5′,8′-diepoxide, which was absent only in the SSD group. As this compound was not identified in either the administered extract or the experimental diets, its presence suggests that β-carotene oxidation occurred during gastrointestinal transit. This metabolite has previously been described as an oxidation product of β-carotene [40]. Likewise, Tao et al. [41] demonstrated that all-*trans*-β-carotene can undergo oxidation, cyclization, and pyrolysis reactions during digestion. In addition, the formation of β-carotene epoxides may be associated with the oxidative conditions of the intestinal lumen or with the metabolic functions of the gut microbiota.

The presence of β-carotene-5,6,5′,8′-diepoxide in all healthy groups and in extract-treated diabetic animals suggests that its formation is not exclusively dependent on the microalgal extract. Instead, it is likely generated through the oxidation of β-carotene derived from both the diet and the administered extract. Conversely, its absence in the SSD group indicates that diabetes may alter carotenoid availability, the intestinal redox environment, or the metabolism of this compound. Nevertheless, supplementation with the microalgal extract appears to have restored conditions favorable for the formation of this metabolite.

Regarding chlorophylls, thirteen degradation products were identified in the cecal contents, demonstrating extensive transformation of these pigments during gastrointestinal transit. As observed in the experimental diets, hydroxypheophytins, pheophytins, and pyropheophytins (peaks 20–26 and 29) were detected in all experimental groups. In contrast, 13^2^-OH-pheophorbide *a* (peak 4) was absent from the groups receiving combined metformin and extract treatment (MLEH and MLED). In addition, three porphyrins were tentatively identified: two were detected in all cecal samples, whereas the third was detected exclusively in the LEH group and in the diabetic groups (SSD, LED, and MLED).

The predominance of chlorophyll derivatives in the cecal samples was expected, as chlorophylls are highly susceptible to chemical, enzymatic, and metabolic modifications throughout gastrointestinal digestion, generating demetallated and/or dephytylated derivatives, hydroxylated compounds, and decarbomethoxylated products through structural alterations of the tetrapyrrolic macrocycle [42]. Despite this, for porphyrins, to date, their occurrence as a product of intestinal chlorophyll metabolism remains poorly documented. Nevertheless, their formation is biologically plausible, as these compounds share the tetrapyrrolic core structure characteristic of chlorophylls [43]. Furthermore, Fernandes et al. [44] reported, for the first time, the occurrence of a porphyrin in samples obtained after simulated gastrointestinal digestion of *S. obliquus*, suggesting that these metabolites may be generated during the gastrointestinal degradation of chlorophylls. In addition, copper porphyrins were also detected in digested samples [45].

Considering that the cecal pH ranges from 5.5 to 6.8, substantially higher than that of the gastric phase (pH < 3.5) (39–41), the presence of these derivatives most likely reflects transformations occurring predominantly in the stomach. In contrast, the origin and metabolic fate of these metabolites within the intestine remain poorly understood. Their detection in the cecal contents suggests either that these compounds are sufficiently stable to persist throughout gastrointestinal transit or that they are continuously generated through microbiota-mediated processes.

### 3.3. Carotenoid and Chlorophyll Quantification

The concentrations of carotenoids and chlorophylls (mg/kg) determined in *S. obliquus*, the diets (CD, HLDD), and the cecal samples from the experimental groups (SSH, LEH, MLEH, SSD, LED, and MLED) are presented in Table 2.

All-*trans*-lutein was the predominant carotenoid in the lipophilic extract of *S. obliquus* (1670.12 mg/kg), the CD (1.28 mg/kg), and the HLDD (1.47 mg/kg). Likewise, all-*trans*-lutein remained the predominant carotenoid in the cecal samples of all experimental groups. This finding is consistent with previous studies demonstrating its limited intestinal absorption, which favors its persistence in the colon [9]. Although carotenoids are not classical fermentative substrates, they may indirectly support microbial activity through their antioxidant properties and their role in maintaining the intestinal redox environment [46,47].

Regarding chlorophylls, although chlorophylls *a* and *b* were the predominant tetrapyrroles in the microalgal extract, pheophytins *a* and *b* were the major chlorophyll derivatives detected in the cecal contents of all experimental groups. This profile indicates that pheophytinization, resulting from the demetallation of the chlorophyll molecule through the loss of the central Mg^2+^ ion, was the predominant transformation occurring during gastrointestinal digestion, whereas dephytylation, which generates pheophorbides, occurred to a lesser extent [48,49].

**Figure 3:**
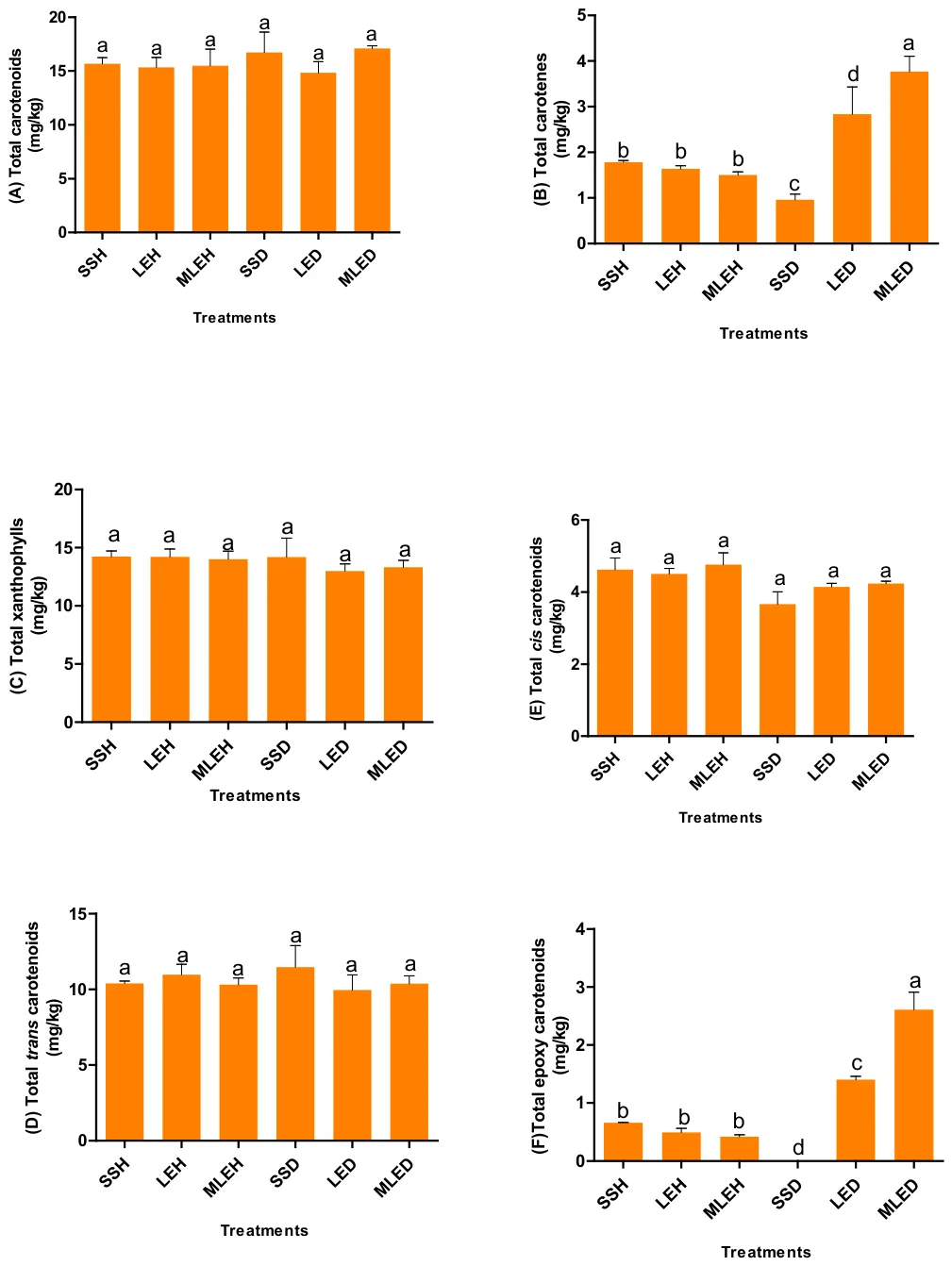
Total contents of carotenoids (**A**), carotenes (**B**), xanthophylls (**C**), *cis*-carotenoids (**D**), *trans*-carotenoids (**E**), and epoxy carotenoids (**F**) in cecal samples. Different letters between columns indicate statistical differences (*p* < 0.05).

**Figure 4:**
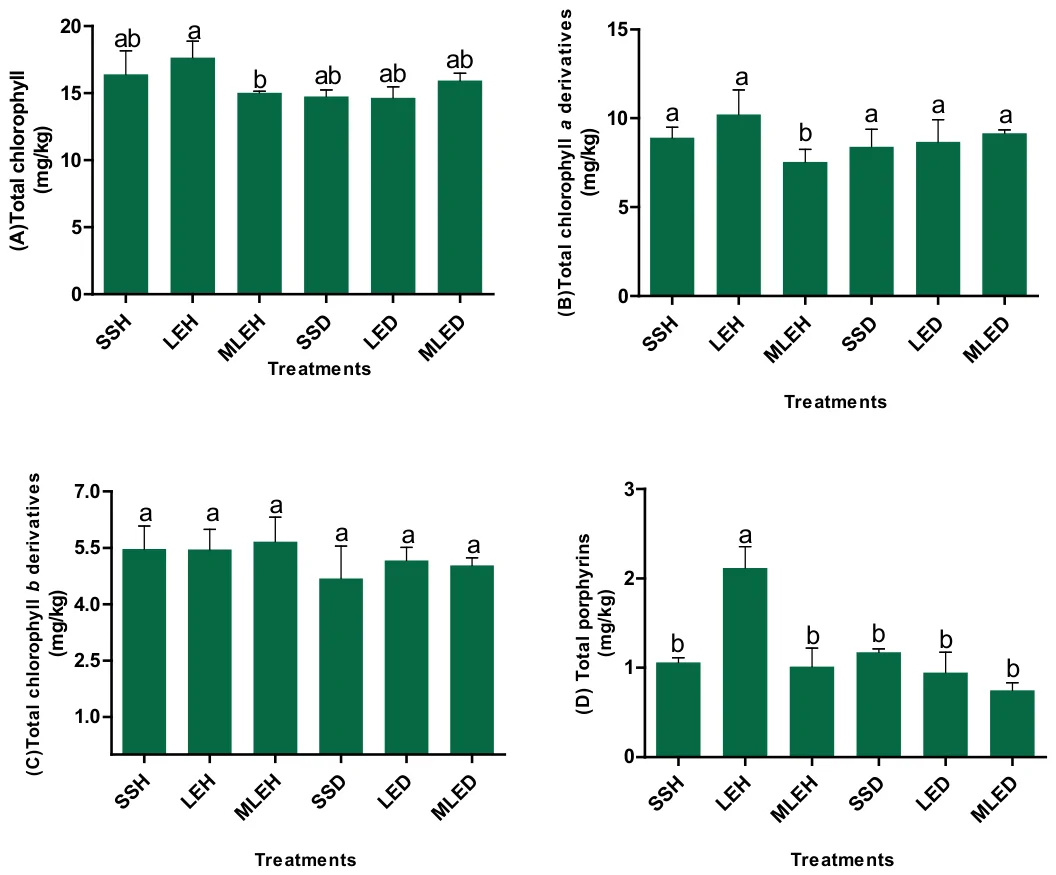
Total contents of chlorophylls (**A**), chlorophyll *a* derivatives (**B**), chlorophyll *b* derivatives (**C**), and porphyrins (**D**) in cecal samples. Different letters between columns indicate statistical differences (*p* < 0.05).

Overall, the lipophilic extract of *S. obliquus* (51,422.51 mg/kg) exhibited a significantly higher total pigment concentration than the diets administered (CD and HLDD), approximately 6-fold and 7-fold higher pigment contents, respectively. These results confirm the potential for microalgal pigment production [50]. In the cecal samples, total pigment concentrations ranged from 29.55 to 34.57 mg/kg, with no statistically significant differences among the experimental groups (*p* > 0.05). These findings indicate that supplementation with the microalgal extract did not result in a detectable increase in the overall pigment content of the cecum. However, individual analyses of the different pigment classes (Figure 3 and Figure 4) revealed treatment-specific changes, indicating that the effects of the extract were associated with the differential distribution of carotenoids and chlorophyll derivatives rather than with an overall accumulation of pigments in this intestinal compartment.

Figure 3 presents the total carotenoid content in the cecal samples, together with their classification into chemically related groups based on structural similarities. Total carotenoids (Figure 3A), xanthophylls (Figure 3C), *cis*-carotenoids (Figure 3D), and *trans*-carotenoids (Figure 3E) did not differ significantly among the experimental groups (*p* > 0.05). In contrast, significant differences were observed for total carotenes (Figure 3B) and epoxycarotenoids (Figure 3F) (*p* < 0.05). The untreated diabetic group (SSD) exhibited the lowest concentrations of these fractions, whereas supplementation with the microalgal extract increased their levels in diabetic animals (LED). The combined treatment with metformin and the microalgal extract resulted in the highest concentrations of carotenes and β-carotene-5,6,5′,8′-diepoxide, suggesting a potential interaction between the two interventions. One possible explanation is that metformin, by attenuating the oxidative stress associated with T2DM, may enhance the stability and/or retention of these carotenoids in the cecal contents [51].

The distribution of total chlorophylls and their derivatives in the cecal samples is presented in Figure 4. Significant differences were observed for total chlorophylls (Figure 4A), chlorophyll *a*-derived compounds (Figure 4B), and porphyrins (Figure 4D), whereas chlorophyll *b*-derived compounds (Figure 4C) remained similar among the experimental groups (*p* > 0.05). The LEH group exhibited the highest concentrations of total chlorophylls and porphyrins, whereas the MLEH group showed the lowest concentration of chlorophyll *a*-derived compounds. The remaining treatments displayed intermediate values.

These results indicate distinct cecal behaviors for carotenoids and chlorophylls. Whereas carotenoids exhibited a more pronounced response to treatment in diabetic animals, chlorophylls and their derivatives displayed a response that was more dependent on the physiological status of the host, with the most evident changes occurring in healthy animals supplemented with the microalgal extract. Overall, the total pigment content in the cecum remained relatively constant, whereas the composition of this pigment pool was substantially remodeled by gastrointestinal digestion, the host’s physiological condition, and supplementation with the microalgal extract. This pattern suggests that these two classes of pigments follow distinct metabolic trajectories after digestion and throughout their persistence within the intestinal environment.

### 3.4. SCFA Levels

**Figure 5:**
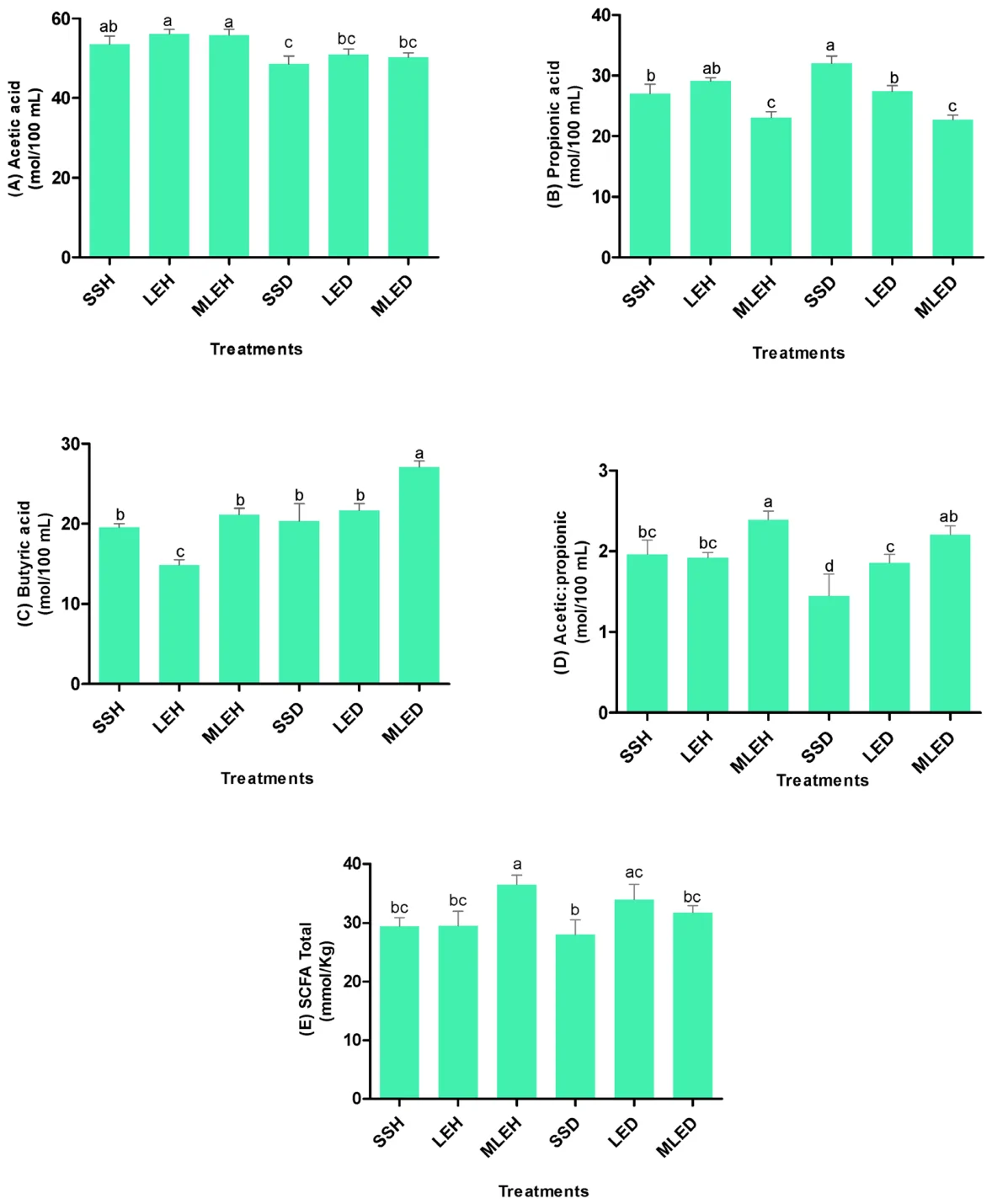
Content of short-chain fatty acids (**A**–**E**) obtained from cecal samples after treatments. SSH: saline solution–Healthy; LEH: lipophilic extract–Healthy; MLEH: metformin + lipophilic extract–Healthy; SSD: saline solution–Diabetic; LED: lipophilic extract–Diabetic; MLED: metformin + lipophilic extract–Diabetic. Different letters between columns indicate statistical differences (*p* < 0.05).

Acetate, propionate, and butyrate are the predominant metabolites generated by colonic microbial fermentation and are widely used to assess gut microbial metabolic function [15,52]. Their concentrations in the cecal contents of the experimental groups are presented in Figure 5.

Acetate was the predominant SCFA, followed by propionate and butyrate, consistent with the typical profile of intestinal fermentation. Acetate concentrations did not differ among the healthy groups (SSH, LEH, and MLEH) or among the treated diabetic groups (LED and MLED) (Figure 5A; *p* > 0.05). In contrast, the untreated diabetic group (SSD) exhibited significantly lower acetate concentrations than the SSH, LEH, MLEH, and LED groups, corroborating previous studies demonstrating the detrimental effects of T2DM on the fermentative activity of the gut microbiota [15]. Notably, supplementation with the microalgal extract (LED), either alone or in combination with metformin (MLED), restored acetate concentrations to levels comparable to those observed in healthy animals. This finding is particularly relevant given the central role of acetate in energy metabolism and appetite regulation [53].

In contrast to the other SCFAs, the SSD group exhibited the highest cecal propionate concentration (Figure 5B), suggesting that T2DM altered intestinal fermentation dynamics. The greater luminal availability of this metabolite should not be interpreted in isolation as a beneficial effect but rather as an indication of fermentative dysregulation associated with diabetes [52,54]. Treatment with the microalgal extract (LED) restored propionate concentrations to values similar to those observed in healthy animals, whereas the combined treatment with metformin (MLED) resulted in a further reduction in this metabolite.

Regarding butyrate (Figure 5C), the LEH group exhibited the lowest concentration, differing significantly from the other treatments, whereas the MLED group showed the highest level. The SSH, MLEH, SSD, and LED groups displayed intermediate concentrations without significant differences among them. This pattern indicates that the effect of the extract on butyrate production depended on both the physiological condition of the host and its combination with metformin. While the extract alone reduced butyrate concentrations in healthy animals, it did not alter butyrate levels in diabetic animals, whereas the combined treatment significantly increased butyrate concentrations exclusively under diabetic conditions. The observed increase may have beneficial physiological implications, as butyrate provides the principal energy source for colonocytes and contributes to intestinal barrier maintenance and immune regulation [53,54].

The reduction in propionate concomitant with the increase in butyrate observed in the MLED group suggests a reorganization of intestinal fermentative metabolism, possibly resulting from the combined effects of metformin and the microalgal extract. This pattern is consistent with evidence that metformin remodels the metabolic activity of the gut microbiota, favoring fermentative pathways associated with butyrate production [55]. In this context, it is plausible that the combination of metformin and the microalgal extract redirected part of the intestinal fermentative metabolism toward butyrate production at the expense of propionate. Because different microbial taxa synthesize propionate through distinct metabolic pathways, including the succinate and propanediol pathways [56], changes in fermentative activity may alter the relative availability of this SCFA without necessarily reflecting changes in its absolute production. Moreover, enhanced intestinal absorption and hepatic utilization of propionate cannot be ruled out, given its role as an important gluconeogenic substrate [57,58]. Therefore, the observed reduction most likely reflects a redistribution of SCFA metabolism rather than a decrease in the absolute production of propionate.

Accordingly, the acetate-to-propionate ratio (Figure 5D) was significantly lower in the SSD group than in the treated groups (*p* < 0.05). Because this ratio is considered an indicator of the functional balance of the gut microbiota [59], its reduction further supports the impairment of intestinal homeostasis associated with T2DM. In contrast, treatment with the microalgal extract, either alone or in combination with metformin, progressively restored this ratio.

With respect to total SCFA concentrations (Figure 5E), the MLEH group exhibited the highest values, differing significantly from the SSD group, which showed the lowest concentrations. The SSH, LEH, LED, and MLED groups displayed intermediate concentrations without significant differences among them. The overall increase in SCFAs observed in treated diabetic animals indicates a partial recovery of intestinal fermentative activity. The relevance of this finding lies in the ability of these metabolites to stimulate the release of glucagon-like peptide-1 (GLP-1) and peptide YY (PYY), which are key regulators of postprandial glycemic homeostasis [53,60]. Although the observed changes in the SCFA profile are consistent with modulation of intestinal fermentative activity, the present study did not directly characterize the gut microbiota. Therefore, microbiota profiling by DNA sequencing would provide valuable information on the microbial taxa involved and help clarify the mechanisms underlying the observed metabolic changes. Future studies integrating microbiome sequencing with metabolomic analyses will further advance the understanding of how microalgal pigments modulate the gut–metabolism axis.

Despite these limitations, the present findings demonstrate that T2DM induces marked alterations in the SCFA profile. Supplementation with the microalgal pigment extract, either alone or in combination with conventional pharmacological therapy, positively modulated SCFA production, shifting the metabolic profile toward that observed in metabolically healthy animals. The restoration of SCFA concentrations and the acetate-to-propionate ratio further highlights the potential of microalgal pigments as a complementary nutritional strategy for the management of T2DM.

### 3.5. Proposed Conceptual Model of Intestinal Pigment Fate

**Figure 6:**
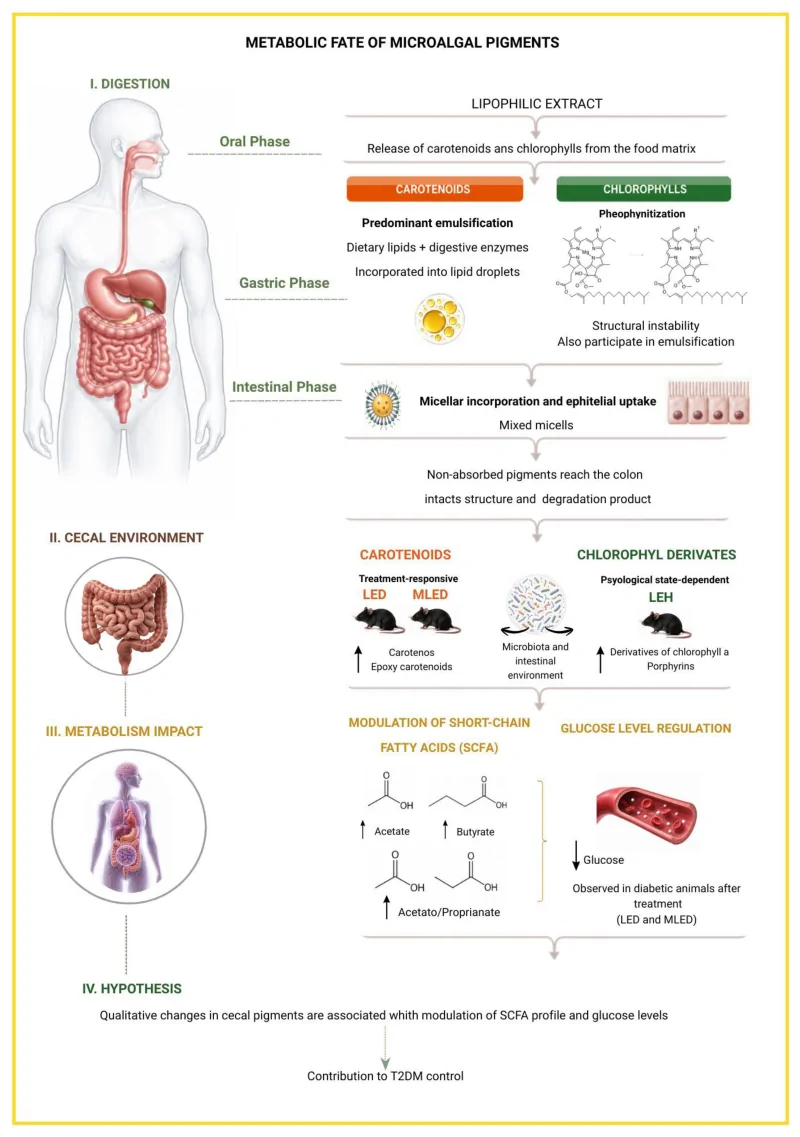
Conceptual model integrating the findings of the present study with the current literature on the gastrointestinal fate of carotenoids and chlorophylls from a microalgal lipophilic extract. (I) After their release from the food matrix, carotenoids are predominantly emulsified and incorporated into mixed micelles, whereas chlorophylls undergo pheophytinization and structural transformations before intestinal uptake; (II) non-absorbed pigments and their degradation products reach the cecum, where they may interact with the gut microbiota. The present study demonstrated a treatment-responsive pattern for carotenoids in diabetic animals and a physiological state-dependent response for chlorophyll derivatives; (III) based on these findings and available evidence, qualitative changes in cecal pigments may contribute to modulation of short-chain fatty acid production and improved glucose regulation. The proposed mechanisms represent a conceptual framework integrating experimental observations with current knowledge.

Based on the integration of the findings obtained in this study with the available literature, a conceptual model was developed to summarize the proposed metabolic fate of carotenoids and chlorophylls during gastrointestinal digestion and their potential interactions with diabetes-associated metabolism. As illustrated in Figure 6, carotenoids and chlorophylls share similar initial stages during digestion but exhibit distinct behaviors throughout the gastrointestinal tract.

During the oral phase, both classes of pigments are released from the food matrix, becoming potentially bioaccessible. In the gastric phase, carotenoids primarily undergo emulsification, a process promoted by dietary lipids and digestive enzymes, resulting in their incorporation into lipid droplets that enhance their stability and facilitate their subsequent incorporation into mixed micelles [61]. In contrast, chlorophylls become structurally unstable under acidic conditions and predominantly undergo pheophytinization through the loss of the central Mg^2+^ ion, although they also participate in the emulsification process [45,62]. During the intestinal phase, the absorption of both pigment classes depends on the efficiency of their incorporation into mixed micelles and their uptake by intestinal epithelial cells [61,62]. The unabsorbed fraction, consisting of intact molecules and their transformation products, reaches the colon, where it remains susceptible to the chemical, enzymatic, and metabolic transformations characteristic of the intestinal environment.

The results of the present study indicate that the host’s metabolic status modulates the intestinal fate of carotenoids and chlorophylls following digestion. In treated diabetic animals (LED and MLED), higher concentrations of carotenes and an epoxycarotenoid—undetected before digestion—were observed, indicating that this class of pigments responded more markedly to the interventions. In contrast, chlorophyll derivatives exhibited a response that was more dependent on the physiological status of the host, as evidenced by the higher concentrations of chlorophyll *a*-derived compounds and porphyrins in the LEH group. Although the mechanisms underlying these differences remain to be fully elucidated, the observed changes in the pigment profile of the cecal contents suggest an association with alterations in intestinal fermentative activity. Collectively, these findings support the hypothesis that microalgal pigments may contribute to modulation of the gut–metabolism axis, thereby promoting the maintenance of metabolic homeostasis in T2DM.

### 3.6. Principal Component Analysis (PCA)

**Figure 7:**
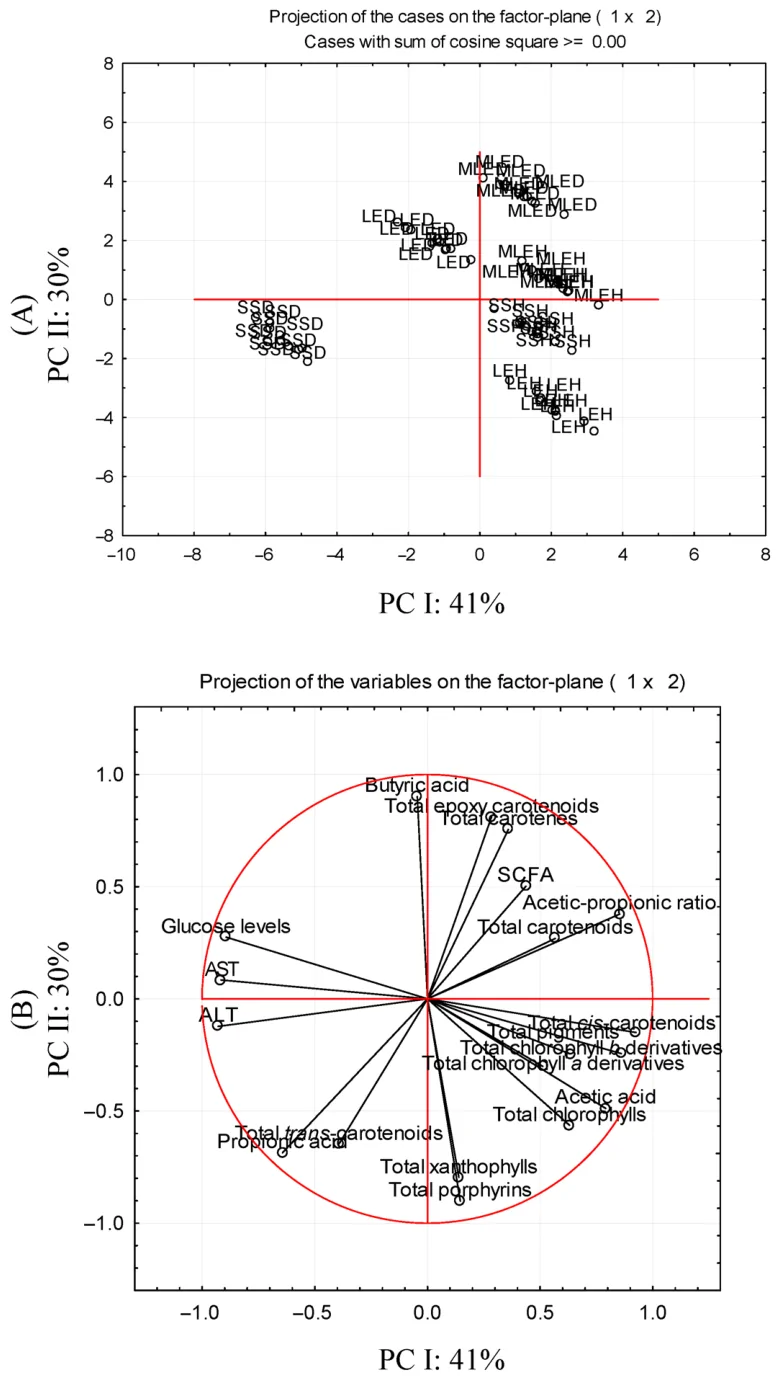
Principal component analysis (PCA) score (**A**) and loading (**B**) plots for principal components 1 (PCI) and 2 (PCII), illustrating the multivariate relationships among cecal pigments, short-chain fatty acids (SCFAs), and glycemic parameters in healthy and diabetic rats.

To complement the conceptual interpretation presented above, a principal component analysis (PCA) was performed to examine the multivariate relationships among pigments, short-chain fatty acids (SCFAs), glycemia, AST, and ALT, providing an integrated assessment of the experimental data (Figure 7).

The PCA explained 71% of the total data variance, with 41% attributed to the first principal component (PC1) and 30% to the second principal component (PC2). The score plot revealed clear discrimination between the experimental groups, indicating that the diabetic condition was a key factor associated with the observed variability (Figure 7A). PC1 drove the primary separation between groups, positioning the untreated diabetic group (SSD) at the negative end of the axis, while the healthy groups (SSH, LEH, and MLEH) clustered predominantly on the positive side. Treated diabetic groups (LED and MLED) showed a shift relative to the SSD group, occupying intermediate positions or positions closer to the healthy groups, suggesting a modulation of the metabolic profile associated with the treatments. PC2 provided additional discrimination between groups, reflecting differences in the set of evaluated variables.

The loading plot showed positive associations of blood glucose, propionic acid, and *trans*-carotenoids with PC1, whereas total pigments, chlorophylls, chlorophyll derivatives, *cis*-carotenoids, acetic acid, the acetate/propionate ratio, and total SCFAs showed a negative association with this component (Figure 7B). AST and ALT behaved similarly to blood glucose, with vectors positioned close together and oriented in the same direction, indicating that these parameters contributed similarly to group discrimination along PC1. Thus, the opposing orientation between blood glucose, AST, and ALT and the majority of pigments and intestinal metabolites suggests an inverse relationship between these sets of variables.

Along PC2, porphyrins, xanthophylls, and propionic acid showed a positive association, while butyric acid, carotenes, and epoxycarotenoids showed negative associations. Furthermore, the proximity and similar orientation of the vectors corresponding to total pigments, chlorophylls, and chlorophyll derivatives indicate a strong association among these variables. In contrast, their opposing trend regarding blood glucose, AST, and ALT levels reinforces the inverse relationship between the pigment profile and these metabolic and biochemical parameters.

Collectively, PCA revealed a relationship between the pigment profile in cecal contents, intestinal fermentation metabolites, and the metabolic and biochemical parameters evaluated. Higher levels of pigments and certain SCFAs, especially acetate, were associated with lower levels of blood glucose, AST, and ALT. Furthermore, the shift of the treated diabetic groups away from the SSD group and toward the profile of healthy animals suggests an attenuation of the alterations associated with the diabetic state. These findings indicate that the lipophilic extract of *S. obliquus*, either alone or in combination with metformin, may contribute to the integrated modulation of intestinal and metabolic alterations related to T2DM.

Finally, these results identify the lipophilic pigments of *S. obliquus* as a source of bioactive compounds to be explored in the context of metabolic alterations associated with T2DM. The effects observed in combination with metformin are particularly relevant, as they raise the possibility of investigating these compounds as a dietary strategy complementary to conventional therapy. Likewise, the relationships between pigment derivatives present in cecal contents and SCFAs provide a basis for future investigations into the role of intestinal metabolism in the observed effects. However, given the preclinical nature and limitations of the present study, these findings should not be directly extrapolated to humans. Further studies on bioavailability, dose–response relationships, safety, and mechanisms of action, followed by clinical trials, will be necessary to determine their applicability as a complementary strategy in T2DM management.

## 4. Conclusions

Overall, this study demonstrates that a lipophilic pigment extract from *S. obliquus* exerts beneficial effects on glycemic metabolism and intestinal fermentative activity in an experimental model of type 2 diabetes mellitus (T2DM). Supplementation, particularly when combined with metformin, attenuated diabetes-induced metabolic alterations by reducing blood glucose levels, partially restoring body weight, and positively modulating the short-chain fatty acid (SCFA) profile. Furthermore, the detection of chlorophyll derivatives, epoxycarotenoids, and porphyrins in cecal contents demonstrates that these pigments undergo extensive metabolic transformations while remaining detectable throughout the gastrointestinal tract. Although a direct association with specific microalgal pigments was not observed, integrated analysis of the results revealed that higher pigment concentrations in cecal contents were associated with a more favorable fermentative profile, characterized by increased SCFA levels (particularly acetate) and lower blood glucose concentrations. The findings also suggest that carotenoids and chlorophylls follow distinct metabolic fates after digestion. While carotenoids showed a more pronounced response to treatment in diabetic animals, chlorophyll derivatives exhibited a response that was more dependent on the host’s physiological state. Finally, these results expand the current understanding of the intestinal fate of microalgal pigments and highlight their potential as a complementary nutritional strategy to modulate the gut-metabolism axis and promote metabolic homeostasis in T2DM.

## Figures and Tables

**Table 1:** Chromatographic and mass spectrometric characteristics of carotenoids and chlorophylls identified by ESI/APCI-HR-TOF-MS^n^.

Peak	Compounds	*t*_R_ (min) ^a^	UV-Vis Maxima (nm)	Elemental Composition	mSigma	Error (ppm)	(M + H)^+^ (*m*/*z*)
1	Chlorophyllide *a*	4.6	432, 666	C_35_H_34_MgN_4_O_5_	16.6	−4.8	615.2482
2	Porphyrin ^b^	4.8	404	nd ^c^	nc ^d^	nc	nd
3	Porphyrin ^b^	5.6	398	nd	nc	nc	nd
4	13^2^-OH-pheophorbide *a*	6.3	410, 666	C_35_H_36_N_4_O_6_	12.1	10.3	609.2705
5	Porphyrin ^b^	6.5	400	nd	nc	nc	nd
6	Pheophorbide *a*	6.9–7.3	410, 666	C_35_H_36_N_4_O_5_	15	4.8	593.2760
7	all-*trans*-neoxanthin	8.4	416, 442, 470	C_40_H_56_O_4_	36.5	−3.7	601.4273
8	13 or 15 *cis*-lutein	8.9	420, 442, 468	C_40_H_56_O_2_	33.0	−1.0	569.4353
9	all-*trans*-violaxanthin	9.6	416, 440, 470	C_40_H_57_O_4_	23.4	−3.5	601.4272
10	13 or 15-*cis*-zeaxanthin	11.0	420, 446, 472	C_40_H_56_O_2_	25.6	4.1	569.4330
11	β-carotene-5,6,5′,8′-diepoxide	11.046	400, 426, 450	C_40_H_56_O_2_	11.8	−2.0	569.4373
12	all-*trans*-lutein	11.7	422, 446, 474	C_40_H_56_O_2_	23.6	−0.1	569.4354
13	9-*cis*-lutein	12.4	420, 442, 468	C_40_H_56_O_2_	36.8	0.9	569.4353
14	Chlorophyll *b*	16.3	464, 650	C_55_H_71_MgN_4_O_6_	41.9	−0.2	907.5218
15	Chlorophyll *b’*	16.8	422, 464, 652	C_55_H_71_MgN_4_O_6_	27.3	1.6	907.5204
16	13^2^-OH-chlorophyll *a*	17.1	430, 664	C_55_H_73_MgN_4_O_6_	12.3	3.5	909.5376
17	9-*cis*-zeaxanthin	17.5	420, 446, 472	C_40_H_56_O_2_	22.5	3.0	569.4369
18	Chlorophyll *a*	18.5	430, 664	C_55_H_72_MgN_4_O_5_	34.9	2.3	893.5451
19	Chlorophyll *a’*	19.3	430, 664	C_55_H_72_MgN_4_O_5_	36.9	1.8	893.5461
20	13^2^-OH-pheophytin *b*	20.5	432, 655	C_55_H_72_N_4_O_7_	25.7	4.3	901.5435
21	13^2^-OH-pheophytin *b’*	20.8	426, 655	C_55_H_72_N_4_O_7_	42.1	−3.2	901.5503
22	13^2^-OH-pheophytin *a*	22.6	410, 664	C_55_H_75_N_4_O_6_	44.5	−0.6	887.5703
23	Pheophytin *b*	23.1	434, 654	C_55_H_37_N_4_O_6_	34.8	3.0	885.5498
24	13^2^-OH-pheophytin *a’*	23.5	410, 664	C_55_H_75_N_4_O_6_	38.1	−1.7	887.5703
25	Pheophytin *a*	25.6	408, 666	C_55_H_75_N_4_O_5_	19.7	−1.3	871.5750
26	Pheophytin *a’*	26.2	408, 666	C_55_H_75_N_4_O_5_	7.9	2.4	871.5709
27	all-*trans*-β-carotene	26.7	425, 452, 478	C_40_H_57_	12.9	3.3	537.4436
28	Pyropheophytin *a*	28.6	410, 664	C_53_H_72_N_4_O_3_	11.2	0.1	813.5664

Pigment identification was performed in *S. obliquus* biomass, experimental diets, and cecal contents. ^a^ Retention time on the C18 column: HPLC; ^b^ tentatively identified; ^c^ not determined; ^d^ nc: not calculated.

**Table 2:** Carotenoids and chlorophylls quantified in microalgae extract, diets, and rat cecal contents (mg/kg dry weight, mean ± SD). Different letters between columns indicate statistical differences (*p* < 0.05).

Peak	Compounds	*S. obliquus* *	CD *	HLDD *	SSH *	LEH *	MLEH *	SSD *	LED *	MLED *
1	Chlorophyllide *a*	358.03 ± 7.16	nd	nd	nd	nd	nd	nd	nd	nd
2	Porphyrin	nd	nd	nd	nd	0.34 ±0.04	nd	0.33 ± 0.08	0.36 ± 0.04	0.27 ± 0.06
3	Porphyrin	nd	nd	nd	0.70 ± 0.45	0.93 ± 0.14	0.26 ± 0.00	0.48 ± 0.06	0.47 ± 0.16	0.34 ± 0.11
4	13^2^-OH-pheophorbide *a*	nd	0.09 ± 0.00	nd	0.67 ± 0.45	1.16 ± 0.18	nd	0.63 ± 0.15	1.04 ± 0.06	nd
5	Porphyrin	nd	nd	nd	0.32 ± 0.20	0.84 ± 0.14	0.72 ± 0.19	0.36 ±0.06	0.34 ±0.01	0.19 ± 0.05
6	Pheophorbide *a*	408.90 ± 16.36	0.13 ± 0.01	0.06 ± 0.00	nd	0.75 ± 0.15	nd	0.19 ± 0.05	0.42 ± 0.11	0.10 ± 0.02
7	all-*trans*-neoxanthin	420.46 ± 8.41	nd	nd	nd	nd	nd	nd	nd	nd
8	*cis*-lutein	302.08 ± 6.04	nd	nd	nd	nd	nd	nd	nd	nd
9	all-*trans*-violaxanthin	203.23 ± 4.06	nd	nd	nd	nd	nd	nd	nd	nd
10	13 or 15 -*cis*-zeaxanthin	nd	0.56 ± 0.07	0.70 ± 0.03	nd	nd	nd	nd	nd	nd
11	β-carotene-5,6,5′,8′-diepoxide	nd	nd	nd	0.66 ± 0.02	0.55 ± 0.05	0.42 ± 0.07	nd	1.77 ± 0.63	2.47 ± 0.37
12	all-*trans*-lutein	1670.12 ± 13.36	1.28 ± 0.11	1.47 ± 0.23	9.62 ± 0.39	9.72 ± 1.19	9.24 ± 0.76	10.52 ± 2.59	8.87 ± 0.71	9.09 ± 0.47
13	9-*cis*-lutein	414.44 ± 12.43	0.14 ± 0.01	0.25 ± 0.02	2.91 ± 0.69	2.80 ± 0.30	3.11 ± 0.42	2.33 ± 0.37	2.72 ± 0.31	2.75 ± 0.06
14	Chlorophyll *b*	5570.54 ± 50.13	nd	nd	nd	nd	nd	nd	nd	nd
15	Chlorophyll *b’*	1357.68 ± 16.29	nd	nd	nd	nd	nd	nd	nd	nd
16	13^2^-OH-chlorophyll *a*	1030.71 ± 20.61	nd	nd	nd	nd	nd	nd	nd	nd
17	*9-cis*-zeaxanthin	145.65 ± 2.91	0.20 ± 0.02	0.23 ± 0.01	1.72 ± 0.10	1.71 ± 0.24	1.66 ± 0.23	1.33 ± 0. 23	1.42 ± 0.16	1.49 ± 0.06
18	Chlorophyll *a*	29,462.16 ± 265.16	nd	nd	nd	nd	nd	nd	nd	nd
19	Chlorophyll *a’*	4211.84 ± 54.75	nd	nd	nd	nd	nd	nd	nd	nd
20	13^2^-OH-pheophytin *b*	nd	0.37 ± 0.02	0.45 ± 0.02	1.00 ± 0.18	nd	0.33 ± 0.00	1.01 ± 0.03	1.17 ± 0.19	1.14 ± 0.07
21	13^2^-OH-pheophytin *b’*	nd	0.39 ± 0.02	0.35 ± 0.01	0.95 ± 0.04	1.33 ± 0.12	1.45 ± 0.16	1.29 ± 0.27	1.05 ± 0.19	1.46 ± 0.09
22	13^2^-OH-pheophytin *a*	nd	0.30 ± 0.02	0.23 ± 0.01	0.95 ± 0.11	0.92 ± 0.26	0.87 ± 0.11	0.86 ± 0.24	1.06 ± 0.20	1.10 ± 0.02
23	Pheophytin *b*	nd	0.77 ± 0.04	0.53 ± 0.06	3.52 ± 0.89	4.04 ± 0.48	4.00 ± 0.39	2.73 ± 0.23	2.74 ± 0.22	2.43 ± 0.09
24	13^2^-OH-pheophytin *a’*	nd	0.13 ± 0.01	0.09 ± 0.01	0.58 ± 0.16	0.72 ± 0.09	0.79 ± 0.12	0.43 ± 0.10	0.54 ± 0.03	0.61 ± 0.05
25	Pheophytin *a*	3163.79 ± 28.47	1.09 ± 0.10	0.65 ± 0.01	4.93 ± 0.14	4.82 ± 0.63	4.36 ± 0.39	3.36 ± 0.84	3.82 ± 0.49	3.85 ± 0.14
26	Pheophytin *a’*	1585.23 ± 20.61	0.74 ± 0.05	0.88 ± 0.03	2.24 ± 0.08	2.35 ± 0.26	2.05 ± 0.19	1.73 ± 0.34	1.98 ± 0.27	2.12 ± 0.05
27	all-*trans*-β-carotene	1117.65 ± 22.35	0.44 ± 0.03	0.38 ± 0.01	1.16 ± 0.00	1.26 ± 0.17	1.08 ± 0.12	0.96 ± 0.24	1.26 ± 0.29	1.30 ± 0.04
28	Pyropheophytin *a*	nd	0.29 ±0.01	0.49 ±0.02	0.50 ± 0.11	0.43 ± 0.13	0.34 ± 0.03	1.34 ± 0.64	1.63 ± 0.48	2.40 ± 0.10
Total		51,422.51 ± 411.38	7.72 ± 0.51 ^c^	6.76 ± 0.27 ^c^	32.47 ± 1.60 ^b^	34.57 ± 3.59 ^b^	30.47 ± 3.13 ^b^	29.55 ± 2.44 ^b^	31.06 ± 3.42 ^b^	33.04 ± 0.76 ^b^

CD: commercial diet; HLDD: high-lipid-density diet; SSH: saline solution–Healthy; LEH: lipophilic extract–Healthy; MLEH: metformin + lipophilic extract–Healthy; SSD: saline solution–Diabetic; LED: lipophilic extract–Diabetic; MLED: metformin + lipophilic extract–Diabetic; nd: not detected; * mg/kg; Different letters between columns indicate statistical differences (*p* < 0.05).

## Data Availability

The original contributions presented in this study are included in the article/Supplementary Materials. Further inquiries can be directed to the corresponding author.

## Supplementary Materials

The following supporting information can be downloaded at: https://www.mdpi.com/article/10.3390/life16091540/s1, Table S1: serum activities of aspartate aminotransferase (AST) and alanine aminotransferase (ALT) in healthy and diabetic rats after 28 days of treatment. Different letters between lines indicate statistical differences (*p* < 0.05).

## Abbreviations

The following abbreviations are used in this manuscript:

APCI	Atmospheric Pressure Chemical Ionization
CD	Commercial Diet
DMF	Dimethylformamide
ESI	Electrospray Ionization
GC-FID	Gas Chromatography–Flame Ionization Detection
GLP-1	Glucagon-Like Peptide-1
HLDD	High-Lipid-Density Diet
HPLC	High-Performance Liquid Chromatography
HPLC-PDA	High-Performance Liquid Chromatography with Photodiode Array Detection
HRQqTOF-MS^2^	High-Resolution Quadrupole Time-of-Flight Tandem Mass Spectrometry
LED	Lipophilic Extract–Diabetic
LEH	Lipophilic Extract–Healthy
MgCO_3_	Magnesium Carbonate
MLED	Metformin + Lipophilic Extract–Diabetic
MLEH	Metformin + Lipophilic Extract–Healthy
NaCl	Sodium Chloride
PCA	Principal Component Analysis
PYY	Peptide YY
SCFAs	Short-Chain Fatty Acids
SD	Standard Deviation
SSD	Saline Solution–Diabetic
SSH	Saline Solution–Healthy
STZ	Streptozotocin
T2DM	Type 2 Diabetes Mellitus
UHPLC	Ultra-High-Performance Liquid Chromatography
UV	Ultraviolet
