# Supplementary material for: Effects of Bioactive Microalgal Pigment Intake on Metabolic Parameters, Cecal Distribution and Short-Chain Fatty Acids in a Diabetic Rat Model
Source: Life (Basel). 2026 Sep 15;16(9):1540. doi: 10.3390/life16091540 (PMC13608300; doi:10.3390/life16091540)
Supplement: Supplementary file 1 [file life-16-01540-s001.zip › life-4517483-supplementary.pdf]

Supplementary Table S1. Serum activities of aspartate aminotransferase (AST) and alanine aminotransferase (ALT) in healthy and diabetic rats after 28 days of treatment. Different letters between lines indicate statistical differences ($p < 0.05$).

Groups	AST (U/L)	ALT (U/L)
SSH	115.15±7.57[c]	59.00±7.50[c]
LEH	124.20±52.96[c]	47.25±1.91[c]
MLEH	115.85±0.07[c]	51.20±8.20[c]
SSD	177.80±42.00[a]	90.10±30.69[a]
LED	145.60±20.36[b]	70.70±45.11[b]
MLED	138.90±5.94[b]	45.55±10.11[c]
